# Potentially inappropriate medication use among older outpatients-prevalence and contributing factors: a retrospective study

**DOI:** 10.1590/1516-3180.2026.3658.08062026

**Published:** 2026-08-14

**Authors:** Meris Esra Bozkurt, Ercument Ozturk

**Affiliations:** I MD. Associated Professor, Department of Internal Medicine, Division of Geriatrics Mersin University, Mersin, Türkiye.; II MD. Associated Professor, Department of Internal Medicine, Division of Geriatrics Mersin City Hospital, Mersin, Türkiye.

**Keywords:** Potentially Inappropriate Medication List, STOPP START Criteria, Aged, Potentially Inappropriate Medication Use, STOPP/START 3, Older Adults

## Abstract

**BACKGROUND:**

Adverse outcomes associated with potentially inappropriate medication (PIM) use are prevalent among older adults.

**OBJECTIVES:**

This study aimed to determine the frequency of PIM use older outpatients and assess its association with chronic comorbidities and geriatric syndromes using the screening tool of older persons’ prescriptions (STOPP) and screening tool to alert to right treatment (START) version 3 criteria.

**DESIGN AND SETTING:**

This retrospective study included patients aged 65 years or older who attended geriatric outpatient clinics between January and June 2024.

**METHODS:**

PIM use was evaluated using the STOPP/START version 3 criteria. Polypharmacy was defined as the use of five or more medications.

**RESULTS:**

Among the 107 patients (median age, 77 years; range, 65-91), 71% (n = 76) used PIM, with a median of two PIMs per patient (range, 0-6). PIM use was significantly more prevalent among patients with polypharmacy (odds ratio [OR] = 3.986; 95% CI: 1.493-10.639; p = 0.006). Analyses based on the STOPP criteria also revealed significant associations of PIM use with dementia (OR = 13.240; 95% CI: 1.504-116.529; p = 0.02), polypharmacy (OR = 4.352; 95% CI: 1.476-12.827; p = 0.008), and a history of falls (OR = 3.131; 95% CI: 1.017-9.634; p = 0.047).

**CONCLUSION:**

Polypharmacy was significantly associated with greater odds of PIM use among older adults. Based on the STOPP version 3 criteria, dementia and a history of falls were also associated with increased PIM use in this population.

## INTRODUCTION

The prevalence of potentially inappropriate medication (PIM) use among older adults ranges from 36.7% to 57.5%, depending on the guidelines applied, with even higher rates typically observed in low-income countries.^1^ ^,^ ^2^ ^,^ ^3^ Over the past two decades, the prevalence of PIM use has doubled.^1^ In older populations, PIM use is associated with adverse outcomes such as falls, hip fractures, increased outpatient visits, and emergency department admissions.^1^ ^,^ ^2^ ^,^ ^3^

The global population is aging rapidly.^3^ Older adults are particularly susceptible to PIM use because of the presence of multiple chronic conditions.^4^ PIM use in this demographic increases the risk of hospital admissions resulting from functional decline.^5^ To mitigate these risks, various guidelines have been established to minimize adverse events associated with PIM use and promote appropriate therapy.^4^ ^,^ ^5^ ^,^ ^6^ These guidelines focus on identifying medications that should be discontinued, detecting inadequate dosing, and recognizing omissions in necessary therapy.^4^ ^,^ ^5^ ^,^ ^6^ The screening tool of older persons’ prescriptions (STOPP) and screening tool to alert to right treatment (START) criteria are the most widely used, and the third version has been recently introduced.^4^ This update incorporates new therapies and serves as an essential reference for optimizing medication safety in older adults. Ongoing evaluation of these guidelines will facilitate further refinements.

Therefore, the present study aimed to examine the prevalence of PIM use among older outpatients and evaluate its associations with chronic diseases and geriatric syndromes using the STOPP/START version 3 criteria.

## MATERIALS AND METHODS

This retrospective study included patients aged ≥ 65 years who attended geriatric outpatient clinics between January and June 2024. The exclusion criteria comprised inability to complete the assessments because of substantial health deterioration (e.g., advanced dementia, organ failure, ongoing cancer treatment, or ischemic sequelae), conditions that prevented the measurement of handgrip strength (e.g., arthritis, neuropathy, plegia, or spasticity), and incomplete data on comorbidities, the numbers of diseases and medications, or geriatric syndromes. The study adhered to the STROBE guidelines.^7^ Ethical approval was obtained from the local university hospital’s ethics committee (approval no. 2024/798).

PIM use was assessed using the STOPP/START version 3 criteria.^4^ The vaccination section was excluded from the assessment. Polypharmacy was defined as the use of five or more medications.^8^

As part of the geriatric assessment, height and weight were measured, and body mass index (BMI) was calculated as weight (kg) divided by height squared (m²).^9^ The recorded variables included age, sex, marital status, smoking status, comorbidities (including diabetes, hypertension, chronic obstructive pulmonary disease [COPD], ischemic heart disease, heart failure, cerebrovascular disease, Parkinson’s disease, kidney disease, dementia, and depression), the numbers of diseases and medications, and geriatric syndromes. The assessed geriatric syndromes included a history of falls, probable sarcopenia, urinary or fecal incontinence, and constipation. Handgrip strength was measured using a dynamometer; and values below 27 kg for men or 16 kg for women were considered indicative of probable sarcopenia.^10^

### Statistical analysis

Categorical data are presented as frequencies and percentages, and continuous data as means ± standard deviations or medians (minimum-maximum) according to their distribution. The Kolmogorov-Smirnov test was used to assess the normality of the data. For univariate analyses, the chi-square test, Mann-Whitney U test, or Student’s t-test was used, as appropriate. Variables that were significant in the univariate analyses were subsequently evaluated using Pearson’s or Spearman’s correlation analysis. Because of the strong correlations among polypharmacy, the number of chronic diseases, and the number of medications, only polypharmacy was included in the multivariable analysis. Multivariable logistic regression analysis was performed, and the results were reported with 95% CI. Statistical significance was set at p < 0.05.

## RESULTS

The median age of the 107 participants was 77 years (range: 65-91 years). The prevalence of PIM use was 71% (n = 76), and the median number of PIMs per patient was 2 (range: 0-6) (Table 1). Table 1 also summarizes the distribution of chronic diseases, including diabetes, hypertension, COPD, congestive heart failure, Parkinson’s disease, chronic renal failure, dementia, and depression, as well as the prevalence of geriatric syndromes such as polypharmacy, falls, probable sarcopenia, urinary or fecal incontinence, and constipation.

**Table 1.** Baseline characteristics of the study population according to the presence of potentially inappropriate medication (PIM) use

	Presence of PIM (n = 76) 71%	Appropriate prescribing (n = 31) 29 %	Total Population (n = 107) 100%	p value
Age^Q^	78 (65-91)	74 (65-87)	77 (65-91)	0.02*
*Sex (n, %)*				
*Male*	21 (27.6%)	10 (32.3%)	31 (29%)	0.632
*Female*	55 (72.4%)	21 (67.7%)	76 (71%)	
Weight (kg)^Q^	72 ± 15.3	75.1 ± 14.5	72.9 ± 15.1	0.342
Height (cm)^Q^	155 (140-183)	155 (143-178)	155 (140-183)	0.823
Body Mass Index (kg/m²)	29.8 ± 6.2	31 ± 6.9	30.1 ± 6.4	0.361
Smoking Status (n, %)				
*Smoker*	4 (5.3%)	4 (12.9%)	8 (7.5%)	0.394
*Quit*	13 (17.1%)	5 (16.1%)	18 (16.8%)	
*Never*	59 (77.6%)	22 (71%)	81 (75.7%)	
Marital Status (n, %)				
*Married*	41 (53.9%)	19 (61.3%)	60 (56.1%)	0.198
*Divorced*	0 (0%)	1 (3.2%)	1 (0.9%)	
*Widowed*	35 (46.1%)	11 (35.5%)	46 (43%)
Chronic disease (n, %)				
*Diabetes mellitus*	27 (35.5%)	8 (25.8%)	35 (32.7%)	0.331
*Hypertension*	56 (73.7%)	21 (67.7%)	77 (72%)	0.535
*COPD*	1 (1.3%)	0 (0%)	1 (0.9%)	0.521
*Ischemic heart disease*	20 (26.3%)	3 (9.7%)	23 (21.5%)	0.057
*Congestive heart failure*	7 (9.2%)	0 (0%)	7 (6.5%)	0.08
*Cerebrovascular disease*	3 (3.9%)	0 (0%)	3 (2.8%)	0.262
*Parkinson’s disease*	4 (5.3%)	0 (0%)	4 (3.7%)	0.193
*Chronic kidney disease*	2 (2.6%)	1 (3.2%)	3 (2.8%)	0.866
*Dementia*	17 (22.4%)	2 (6.5%)	19 (17.8%)	0.051
*Depression*	14 (18.4%)	1 (3.2%)	15 (14%)	0.04*
Polypharmacy (n, %)	64 (84.2%)	15 (48.4%)	79 (73.8%)	< 0.001*
Number of chronic diseases^Q^	3 (0-13)	2 (0-5)	3 (0-13)	0.002*
Number of chronic drugs^Q^	7 (0-16)	3 (0-10)	6 (0-16)	< 0.001*
Geriatric syndromes (n, %)				
*Falls*	26 (34.2%)	5 (16.1%)	31 (29%)	0.061
*Probable sarcopenia (HGS/27*-*16 kg)*	33 (43.4%)	11 (35.5%)	44 (41.1%)	0.449
*Urinary incontinence*	42 (55.3%)	11 (35.5%)	53 (49.5%)	0.063
*Faecal incontinence*	2 (2.6%)	0 (0%)	2 (1.9%)	0.362
*Constipation*	10 (13.2%)	5 (16.1%)	15 (14%)	0.688

BMI: body mass index; COPD: chronic obstructive pulmonary disease; HGS: handgrip strength; PIM: potentially inappropriate medication use. Data are presented as the mean ± standard deviation, median (interquartile range), or number (percentage) as applicable. ^Q^ Data as presented as the median (interquartile range). * Significant p values.

According to the START criteria, the prevalence of PIM use was 29% (n = 31). The medication groups most involved included oral bisphosphonates, acetylcholinesterase inhibitors, and memantine, along with the underdosing of antiplatelet and anticoagulant agents.

Using the STOPP criteria, the prevalence of PIM use was 63.6% (n = 68). The medications or medication classes most frequently implicated were nootropics used to treat dementia (e.g., ginkgo biloba and piracetam), nonsteroidal anti-inflammatory drugs, muscle relaxants, antiplatelet agents, anticoagulants, and proton pump inhibitors.

Univariate analysis showed significant associations between PIM use and age (p = 0.02), depression (p = 0.04), polypharmacy (p < 0.001), number of chronic diseases (p = 0.02), and number of chronic medications (p < 0.001) (Table 1). In the multivariable analysis, only polypharmacy (odds ratio [OR] = 3.986; 95% CI: 1.493-10.639; p = 0.006) remained statistically significant; the associations with age and depression were not significant (Table 2).

**Table 2.** Multivariable analysis of factors associated with PIM use

	p	OR	95% Cl	
			Lower	Upper
Age	0.09	1.063	0.99	1.141
Depression	0.188	4.193	0.497	35.354
Polypharmacy	0.006*	3.986	1.493	10.639

CI: confidence interval; OR: Odds ratio; PIM: potentially inappropriate medication use. * Significant p values.

Univariate analysis based on the STOPP criteria showed significant associations between PIM use and age (p = 0.005), dementia (p = 0.002), depression (p = 0.045), polypharmacy (p < 0.001), the number of chronic diseases (p = 0.004), the number of chronic medications (p < 0.001), and a history of falls (p = 0.019). No significant associations were found with sex (p = 0.895), weight (p = 0.067), height (p = 0.719), BMI (p = 0.105), smoking status (p = 0.28), marital status (p = 0.407), diabetes (p = 0.452), hypertension (p = 0.356), COPD (p = 0.447), ischemic heart disease (p = 0.098), heart failure (p = 0.208), cerebrovascular disease (p = 0.909), Parkinson’s disease (p = 0.628), chronic kidney disease (p = 0.909), probable sarcopenia (p = 0.406), urinary incontinence (p = 0.597), fecal incontinence (p = 0.208), or constipation (p = 0.758).

In the multivariable analysis based on the STOPP criteria, PIM use remained significantly associated with dementia (OR = 13.240; 95% CI: 1.504-116.529; p = 0.02), polypharmacy (OR = 4.352; 95% CI: 1.476-12.827; p = 0.008), and a history of falls (OR = 3.131; 95% CI: 1.017-9.634; p = 0.047) (Table 3).

**Table 3.** Multivariable analysis of factors associated with PIM use based on the STOPP criteria

	p	OR	95% Cl	
			Lower	Upper
Age	0.167	1.050	0.980	1.126
Dementia	0.020*	13.240	1.504	116.529
Depression	0.151	3.351	0.644	17.439
Polypharmacy	0.008*	4.352	1.476	12.827
Falls	0.047*	3.131	1.017	9.634

CI: confidence interval, OR: odds ratio, PIM: potentially inappropriate medication use. * Significant p values.

## DISCUSSION

In this study, the prevalence of PIM use among older outpatients was 71%. PIM use was significantly associated with polypharmacy. In the analysis based on the STOPP criteria, PIM use was significantly associated with dementia and a history of falls.

A meta-analysis by Tian et al.,^1^ which included 94 studies comprising 371.2 million individuals aged ≥65 years across 17 regions, reported a PIM prevalence of 36.7%. The studies in the meta-analysis applied various assessment criteria, including multiple versions of the Beers and the STOPP/START criteria, but did not apply the recently introduced STOPP/START version 3 criteria or the 2023 Beers criteria.^1^ The higher prevalence observed in the present study (71%) may be attributable to the use of the updated STOPP/START version 3 criteria.

A cohort study by Ortonobes et al.^11^ including 674 inpatients aged ≥65 years assessed PIM use during hospitalization using the STOPP/START version 2 criteria and found a prevalence of 73.1%. Although Ortonobes et al. used an earlier version of the criteria and a pharmacologist’s perspective, their findings were comparable to those of the present study, likely due to the substantial overlap between the two versions. In contrast, Andrade et al. applied the local European Union (7)-PIM criteria to 96 institutionalized older adults and reported a higher PIM prevalence of 86%. This difference may be attributable to differences in both the screening criteria and the patient populations.^12^

Multiple previous studies have consistently reported an association between PIM use and polypharmacy, regardless of the screening tool used.^13^ ^,^ ^14^ ^,^ ^15^ ^,^ ^16^ For example, Kojima et al.^13^ used the STOPP for Japanese (STOPP-J) criteria to screen 67,169 individuals aged ≥65 years and identified an association between polypharmacy and PIM use. Similarly, Hagiwara et al.^14^ conducted a cross-sectional study of 67,531 older adults receiving long-term care and reported a comparable association using the STOPP-J criteria. Kelleci et al.^15^ also observed this association among 700 older outpatients assessed using the Beers criteria.

Assessments using the STOPP criteria identify PIMs more frequently than those using the START criteria, regardless of the version used. The results of the present study are consistent with these findings.^15^ ^,^ ^16^ ^,^ ^17^ ^,^ ^18^

Consistent with the present findings, previous studies have identified associations between PIM use assessed using the STOPP criteria and a history of falls, regardless of the STOPP/START version used.^19^ ^,^ ^20^ ^,^ ^21^

The present findings are also consistent with those of previous studies that demonstrated an association between PIM use, as determined using the STOPP criteria, and dementia.^22^ ^,^ ^23^ ^,^ ^24^

A key strength of this study is the application of the recently introduced STOPP/START version 3 criteria and the performance of assessments by geriatric specialists. However, limitations include its retrospective design and the lack of data on vaccination status.

## CONCLUSION

Polypharmacy was significantly associated with PIM use in older adults. Medication regimens should be regularly reviewed to identify PIMs and determine whether they can be safely discontinued, particularly among patients with polypharmacy, a history of falls, or dementia.

## Data Availability

The data used in this study contains sensitive or private information and cannot be shared publicly in accordance with the Ethics Committee of Mersin University. Data are available from the authors upon approval by the relevant committee.
